# Variations in Essential Oils from the Leaves of *Cinnamomum bodinieri* in China

**DOI:** 10.3390/molecules28093659

**Published:** 2023-04-23

**Authors:** Chao Fu, Xinliang Liu, Qian Liu, Fengying Qiu, Jindong Yan, Yueting Zhang, Ting Zhang, Jianan Li

**Affiliations:** 1 College of Forestry, Central South University of Forestry and Technology, Changsha 410004, China; plantfuchao@163.com (C.F.); yjd0731@outlook.com (J.Y.); 2 Camphor Engineering and Technology Research Centre of National Forestry and Grassland Administration, Jiangxi Academy of Forestry, Nanchang 330032, China; 13611574029@163.com (X.L.); lq_lky18670223641@126.com (Q.L.); qiufengying1@126.com (F.Q.);

**Keywords:** *Cinnamomum bodinieri*, essential oil (EO), composition, chemotypes

## Abstract

*Cinnamomum* plants are rich in natural essential oils, which are widely used as materials in the fragrance, insecticidal, antibacterial agent, pharmaceutical, and food industries; however, few studies have investigated the essential oil components of *Cinnamomum bodinieri*. Therefore, this study investigated the diversity of essential oils from the leaves of 885 individual *C. bodinieri* plants across 32 populations in five provinces. Essential oils were extracted by hydrodistillation, and then qualitative and quantitative analyses of the compounds were performed by GC-MS and GC-FID. A total of 87 chemical constituents were identified in the essential oils, including 33 monoterpenes, 48 sesquiterpenes, and six other compounds. The average oil yield was 0.75%, and individual oil yields ranged from 0.01% to 4.28%. A total of 16 chemotypes were classified according to variations in the essential oil chemical constituents of *C. bodinieri*, among which the camphor-type, citral-type, and eucalyptol-type were dominant. Moreover, the borneol-type, cymol-type, elemol-type, methylisoeugenol-type, and selina-6-en-4-ol-type were reported in *C. bodinieri* for the first time. The yield and principal components of the essential oils were mainly affected by altitude, temperature, and sunshine duration, among which altitude had the most significant effect; thus, low-altitude areas are more suitable for the synthesis and accumulation of essential oils. Based on the different characteristics of the essential oils in the leaves of *C. bodinieri*, several excellent populations and individuals were identified in this study. Moreover, the findings provide a foundation for breeding superior varieties and studying essential oil biosynthesis mechanisms in the future.

## 1. Introduction

*Cinnamomum bodinieri* is an evergreen broad-leaved tree of the *Lauraceae* that is distributed only in limestone or karst areas in the western Hunan, Guizhou, Hubei, Sichuan, and eastern Yunnan provinces in China [1]. The branches and leaves of *Cinnamomum* plants are rich in natural essential oils, which can be widely used as materials in the fragrance, insecticidal, antibacterial agent, pharmaceutical, and food industries [2,3,4]. At present, *Cinnamomum camphora* is widely used in the essential oil industry and has been investigated to determine whether *Cinnamomum* plants can be cultivated in forests as a raw material for the production of certain spices; however, the development and application of essential oils from other *Cinnamomum* plants are relatively limited [5]. Compared with *C. camphora*, *C. bodinieri* has the advantages of larger biomass, stronger stress resistance, and more rapid growth, which are important for the development and utilisation of natural *Cinnamomum* essential oils.

Current research on *C. bodinieri* is mainly focused on the stress resistance of the plant and breeding technology [6,7,8,9]. However, few studies have reported on the essential oils of *C. bodinieri.* Relevant studies have extracted and analysed the essential oils from the leaves of *C. bodinieri* and found that they are mainly composed of citral and have strong antioxidant biological activity [10]. The essential oils of plants have complex and diverse compositions, and their synthesis, accumulation, and transformation are affected by genetic and environmental factors [11,12]. Moreover, the chemical composition of essential oils from different individuals of the same species may vary greatly; for example, the essential oil of *C. camphora* has a variety of chemotypes, such as camphor, borneol, and linalool [13,14].

However, the leaf essential oils of only a small number of individuals of *C. bodinieri* have been analysed, and the samples were not collected over a large area. Moreover, relevant reports on the diversity of essential oil from the leaves of *C. bodinieri* are not available, and many new chemotypes of the essential oils of *C. bodinieri* remain to be discovered. Therefore, this study investigated 32 natural populations of *C. bodinieri* in its main distribution areas in China, such as Hunan, Hubei, Guizhou, Sichuan, and Yunnan. In total, 885 *C. bodinieri* leaf samples were collected. The yield, chemical composition, and chemotypes of the essential oils and influential environmental factors were analysed to explore the diversity of the oils and determine the law of population geographic variations. The results provide an important theoretical basis for the breeding, development, and utilisation of *C. bodinieri* essential oils.

## 2. Results

### 2.1. Variation in the Essential Oil Yield

**Figure 1:**
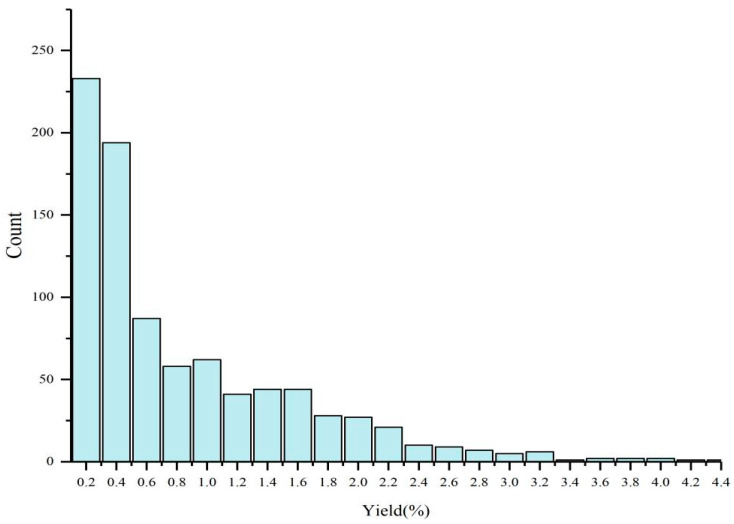
Frequency distribution of the essential oils of *C. bodinieri*.

Essential oils from 885 *C. bodinieri* individuals from 32 populations were extracted and analysed. A frequency distribution map was created using 0.2% as the distribution interval (Figure 1). The results showed that the individual *C. bodinieri* decreased gradually with the increase in the oil yield interval, and the individual *C. bodinieri* oil yield interval was mainly concentrated in the two intervals below 0.4. The largest number of individuals of *C. bodinieri* was 233 in the 0–0.2 interval, followed by 194 in the 0.2–0.4 interval. A total of 427 were found in the first two intervals, which represented 48.2% of the total number. Compared with the previous two intervals, the number of individuals in the 0.4–2.0 interval decreased significantly and ranged from 20 to 90, and the number of individuals in the 2.0–3.0 interval was 5 to 20. The oil yield of a few individuals could exceed 3.0. The highest oil yield could reach 4.28%, while the lowest oil yield was only 0.01%, which represented a difference of 428 times. The great differences between individuals indicate that *C. bodinieri* leaves are naturally extremely rich in essential oil genetic resources, thus indicating that this plant has great potential for breeding.

**Figure 2:**
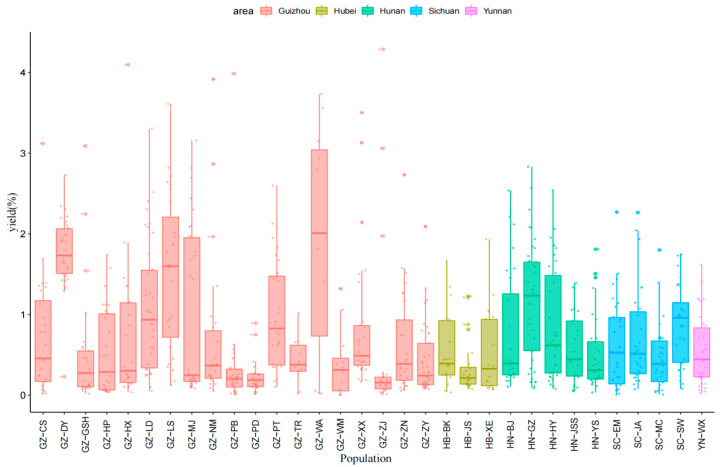
Yield of essential oil from the leaves of *C. bodinieri* from different communities.

The yield of the leaf essential oils of different populations of *C. bodinieri* in different regions was statistically analysed (Figure 2). The average oil yield was 0.75% and the results showed that significant differences occurred between the different populations of *C. bodinieri*. The average oil yield in Guizhou was 0.80%, among which GZ-WA (1.95%), GZ-DY (1.77%), GZ-LS (1.52%), GZ-LD (1.09%), and GZ-MJ (1.02%) had higher oil yields. The average oil yield in Hubei province was 0.47%, and the oil yield of various groups was ordered as follows: HB-BK (0.56%) > HB-XE (0.52%) > HB-JS (0.35%). The average oil yield in Hunan was 0.77%, and the oil yield of various groups was ordered as follows: HN-GZ (1.15%) > HN-HY (0.85%) > HN-BJ (0.78%) > HN-JSS (0.57%) > HN-YS (0.51%). The average oil yield in Sichuan was 0.67%, and the oil yield of various groups was ordered as follows: SC-SW (0.87%) > SC-JA (0.71%) > SC-EM (0.63%) > SC-MC (0.47%). The oil yield in Yunnan (YN-WX) was 0.55%.

### 2.2. Composition and Correlations of the Main Chemical Constituents

**Figure 3:**
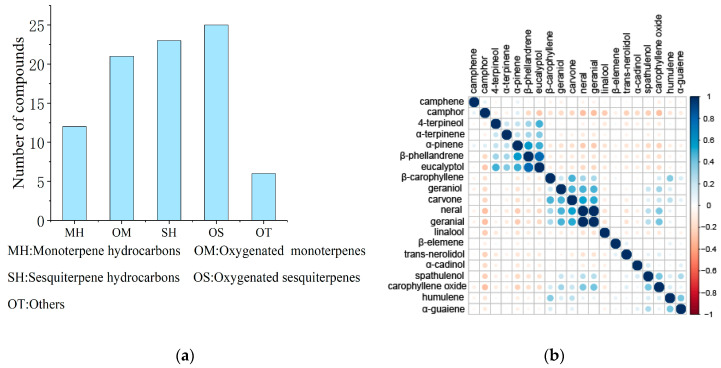
(**a**) Classification of the chemical components of the essential oils in the leaves of *C. bodinieri.* (**b**) Correlation of the chemical components of the essential oils in the leaves of *C. bodinieri*.

A total of 87 compounds were identified by statistical analysis of the leaf components of wild *C. bodinieri* populations, and they included 12 hydrocarbon monoterpenes, 21 oxygen-containing monoterpenes, 23 hydrocarbon sesquiterpenes, 25 oxygen-containing sesquiterpenes, and six other compounds (Figure 3a).

Correlations between the 20 chemical components with the highest frequencies were analysed (Figure 3b). The results showed that neral and geranial in *C. bodinieri* essential oil were significantly correlated and often found together in the essential oils. Β-Phellandrene, eucalyptol, and 4-terpineol were significantly positively correlated; carvone, trans-nerolidol, and β-carophyllene were significantly positively correlated; and spathulenol and carophylene oxide were significantly positively correlated. Camphor was negatively correlated with eucalyptol, linalool, neral, geranial, and trans-nerolidol, and linalool was negatively correlated with eucalyptol, neral, and geranial.

### 2.3. Chemotype Classification and Principal Component Analysis

**Figure 4:**
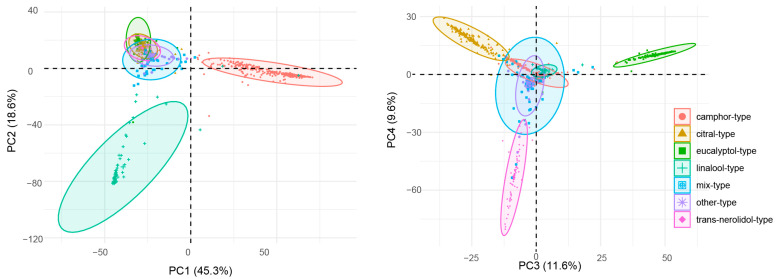
Principal Component Analysis and Chemical Type Classification of the Essential Oil from the Leaves of *C. bodinieri*.

The chemical composition of the essential oils from 885 *C. bodinieri* plants from 32 populations was statistically analysed based on a principal component analysis (Figure 4). The first four principal components (PC1, PC2, PC3, and PC4) accounted for 45.3%, 18.6%, 11.6%, and 9.6% of the variation, respectively, which represented 85.1% of the total variation. Camphor was clearly separated from linalool based on PC1 and PC2; camphor was positively correlated with PC1 and negatively correlated with PC2, and linalool was negatively correlated with PC1 and PC2. PC3 and PC4 could separate eucalyptol, trans-nerolidol, and citral, in which eucalyptol was positively correlated with PC3 and PC4, trans-nerolidol was negatively correlated with PC3 and PC4, and citral was negatively correlated with PC3 and positively correlated with PC4. The principal component analysis revealed that 885 individual *C. bodinieri* plants could be divided into seven types, namely, camphor-type, citral-type, eucalyptol-type, linalool-type, trans-nerolidol-type, mixed-type (principal component content <30%), and other chemotypes.

### 2.4. Chemotypes of C. bodinieri Essential Oil

According to the relative principal component analysis, all the essential oils were divided into 16 chemotypes (Table 1). Camphor-type and citral-type accounted for the greatest amounts at 333 and 160, which represented 37.63% and 18.08% of the population, respectively, and they were followed by eucalyptol-type, linalool-type, mix-type, and trans-nerolidol-type at 101 (11.41%), 82 (9.27%), 73 (8.25%), and 65 (7.34%), respectively.

A significant difference was observed in the oil yields of some chemotypes of *C. bodinieri* (*p* < 0.05). The camphor-type had the highest oil yield, with an average of 1.18%, followed by linalool-type, eucalyptol-type, and selina-6-en-4-ol-type, with averages of 1.06%, 0.73%, 0.95%, and 0.73%, respectively. The safrole-type oil yield was the lowest (0.08%). The oil yields and relative principal component values of different individuals of the same chemotype were significantly different. For example, the oil yield of the camphor-type ranged from 0.02% to 4.28%, and the relative principal component value was 30.42–98.52%; the oil yield of the linalool-type ranged from 0.01 to 2.73%, and the relative principal component value was 31.39–97.14%; and the oil yield of the citral-type ranged from 0.02 to 1.51%, and the relative principal component value was 31.77–89.12%.

Although the chemotypes of the essential oil of *C. bodinieri* were mainly camphor-type, linalool-type, citral-type, eucalyptol-type, trans-nerolidol-type, and mixed-type, other chemotypes were observed. These chemotypes were very rare and found in less than 20 plants, while some chemotypes were only found in one plant. However, most of these chemotypes were unique chemical types discovered for the first time in *C. bodinieri*, especially in certain individuals with high relative principal component loadings, including HN-YS-17 (α-cadinol content of 62.79%), HN-JSS-25 (cymol content of 67.71%), HN-JSS-09 (elemol content of 74.29%), HB-JS-11 (methyleugenol content of 94.86%), YN-WX-13 (methylisoeugenol content of 88.81%), GZ-ZJ-16 (safrole content of 77.09%), and HB-BK-16 (selina-6-en-4-ol content of 67.15%).

**Figure 5:**
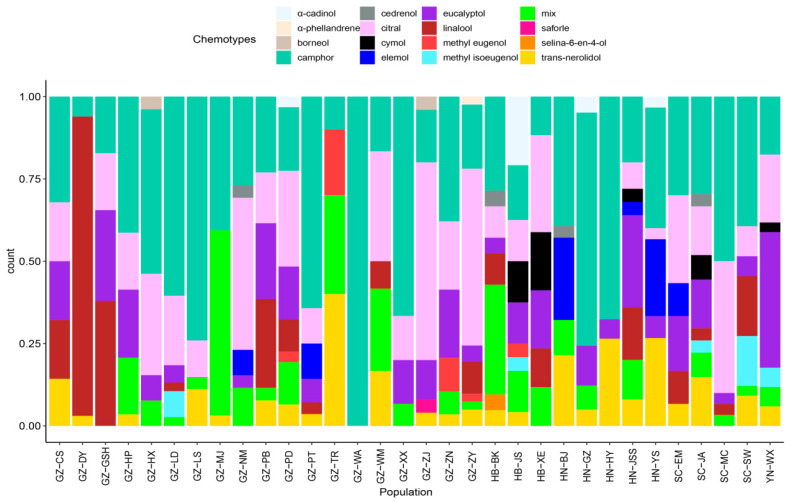
Distribution of chemical types in different communities of *C. bodinieri*.

The proportion of different chemotypes in each *C. bodinieri* population was analysed (Figure 5). The results showed that the camphor-type existed in all investigated populations and was dominant in GZ-WA, GZ-XX, HN-GZ, HN-HY, GZ-LS, and GZ-LD. Therefore, the camphor-type was the most abundant and widely distributed chemotype in the *C. bodinieri* populations. Second, the main chemotype of GZ-DY and GZ-GSH was the linalool-type, which accounted for more than 90%, although a few individual plants reached 97%. In addition, the oil yield of these populations reached 2.1%. The GZ-NM, GZ-ZJ, and GZ-ZY populations were dominated by the citral-type, GZ-TR by the trans-nerolidol-type, and GZ-MJ was dominated by the mixed-type. In addition, the chemotypes of the GZ-WA, GZ-DY, and HN-HY populations were relatively small, with only 1–3 chemotypes in the populations, while chemotypes of HB-BK, HB-JS, GZ-ZY, GZ-XE, HN-JSS, and GZ-PD were more abundant

### 2.5. Correlation between C. bodinieri Essential Oils and Environmental Factors

The correlation between the oil yield of *C. bodinieri* leaves and the relative contents of the seven main chemotypes and geographical environmental factors was analysed (Table 2). The results showed that the oil yield was significantly negatively correlated with altitude and positively correlated with annual rainfall and average temperature. The relative camphor, eucalyptol, linalool, and citral contents were significantly negatively correlated with altitude. The relative citral content was positively correlated with the annual average temperature. The relative elemol, cymol, and camphor contents were positively correlated with the annual average sunshine length.

Comprehensive analysis showed that latitude and longitude were not the main factors affecting the essential oil of *C. bodinieri*, although altitude, temperature, and sunshine length had significant effects and altitude difference had the most obvious effect. The yield of *C. bodinieri* essential oil and relative content of the main components, such as camphor-type, citral-type, and linalool-type, were significantly higher in low-altitude areas than high-altitude areas.

## 3. Discussion

*Cinnamomum* plants usually contain natural essential oils in their roots, stems, and leaves, and some of these species have been widely cultivated as important spice trees, thus forming a complete industrial chain that integrates planting, harvesting, extraction, and processing [15,16]. The essential oil of *Cinnamomum* plants has abundant chemical components and thus has been studied by many domestic and foreign scholars. Researchers have studied the diversity of essential oils from *Cinnamomum parthenoxylon* leaves and classified the oils into 14 chemotypes. The dominant chemotypes are the camphor-type and linalool-type [17]. The main components in the essential oil of *Cinnamomum zeylanicum* leaves are E-cinnamaldehyde and eugenol [18]; the main component in the essential oil of *Cinnamomum longipaniculatum* is eucalyptol [19]; and the main component of the essential oil of *Cinnamomum pauciflorum* leaves is safrole [20].

In this study on the diversity of leaf essential oils from 32 populations of *C. bodinieri*, essential oils were divided into 16 chemotypes. The essential oil chemotype contents of *C. bodinieri* are among the most abundant of *Cinnamomum* plants, and the dominant chemotypes are camphor-type, citral-type, and eucalyptol-type. Camphor is a common chemical component of *Cinnamomum* plants [21], the camphor-type was found in the largest number of individuals among the *C. bodinieri* populations. The individual oil yield varied greatly, with the highest oil yield of 4.28%, which was significantly higher than that reported for *C. camphora* (3.46%) [22]. Citral essential oil is mainly found in the fruit of *Litsea cubeba* and rarely occurs as the main component in the essential oils of *C. camphora*, *C. longipaniculatum*, *C. parthenoxylon*, and other *Cinnamomum* plants [23,24]; however, 160 citral-type individuals were found in the currently studied *C. bodinieri* population, thus accounting for 16.7% of the total number of individuals, which was significantly higher than that of other *Cinnamomum* species. Eucalyptol mainly exists in eucalyptus plants and has many functions, such as anti-inflammatory, analgesic, antibacterial, and tumour cell inhibition; thus, it is primarily used in industrial raw materials and medicine [25,26]. The relative content of eucalyptus in the essential oil of *C. bodinieri* was as high as 67.09%, and the highest oil yield was 2.86%, which was higher than that of most eucalyptus plants. Therefore, these three excellent chemotypes should be screened in *C. bodinieri* to determine the potential use of this species in forests to generate spice raw material. In addition, studies on *C. camphora, C. parthenoxylon*, *Cinnamomum tenuipilum*, and *Cinnamomum pauciflorum* have reported the dominance of the borneol-type, cymol-type, elemol-type, and methylisoeugenol-type [27]; however, this study provides the first report of these chemotypes in *C. bodinieri*.

The essential oils of *Cinnamomum* are primarily monoterpenes, sesquiterpenes, and phenylpropanoids. Monoterpene biosynthesis in plants mainly originates from the methylerythritol phosphate pathway (MEP), sesquiterpene biosynthesis mainly originates from the mevalonate pathway (MVA), and phenylpropanoid synthesis mainly originates from the shikimate pathway [28]. A total of 87 chemical constituents were detected in the essential oil of *C. bodinieri* leaves, including 33 monoterpenes, 48 sesquiterpenes, and six other compounds. These chemical components are related to each other, indicating the complexity of the formation mechanism of the essential oils of *Cinnamomum*. An in-depth study of the genome and transcriptome of *Lauraceae* species, such as *C. camphora*, *L. cubeba*, and *C. parthenoxylon*, has made important breakthroughs in the study of terpenoid biosynthesis pathways and mechanisms, such as linalool, camphor, eucalyptol, trans-nerolidol, and citral [29,30,31]. Few studies on *Cinnamomum* have investigated terpenoids, such as α-phellandrene and cymol, and phenylpropanoid compounds, such as safrole and methylisoeugenol; therefore, the discovery of new chemotypes in *C. bodinieri* provides an important basis for further studies on the biosynthesis mechanism of essential oils in *Cinnamomum.*

Significant differences were observed in the essential oils of *C. bodinieri* leaves among the different populations. Most populations were dominated by the camphor-type; GZ-DY and GZ-GSH were dominated by the linalool-type; GZ-NM, GZ-ZJ, and GZ-ZY were dominated by the citral-type; and YN-WX was dominated by the eucalyptol-type. The HB-BK, HB-JS, GZ-ZY, GZ-XE, HN-JSS, and GZ-PD communities are abundant and can be used as candidate populations for breeding new chemotypes. Moreover, the difference in essential oils between individuals in the *C. bodinieri* populations are also very significant, and this phenomenon of significant differences within and between populations has also been noted in studies on the diversity of leaf essential oils of other *Cinnamomum* plants [32,33]. Therefore, the diversity between populations and individuals should be fully considered when selecting essential oil resources from *Cinnamomum*.

The synthesis and accumulation of plant essential oils are affected by multiple genetic and environmental factors, including altitude, longitude, latitude, temperature, and rainfall [34,35]. The essential oil yields of *Thymus carmannicus* and *Oliveria decumbens* gradually decreased with increasing altitude [36,37]. The accumulation of *Lavandula latifolia* essential oil was mainly affected by latitude and rainfall [38]. The correlation analysis between the essential oils of *C. bodinieri* leaves and environmental factors showed that altitude, sunshine duration, and annual average temperature had the greatest effects, while longitude and latitude had weaker effects. The climate at low altitudes in southern China is warm and humid, which can significantly increase the yield of volatile oil from *C. bodinieri* leaves. Although the natural distribution of *C. bodinieri* is mainly in the southwest of China, according to its adaptability to environmental factors, it can be introduced to the southern and southeastern low-altitude areas of China, such as Jiangxi, Fujian, and Guangdong, to improve the yield of *C. bodinieri* essential oil.

## 4. Materials and Methods

### 4.1. Sampling of Plant Materials

From July 2019 to August 2021, 32 natural populations of *C. bodinieri* were collected from the five provinces of Guizhou, Hunan, Hubei, Sichuan, and Yunnan. A total of 511, 62, 158, 120, and 34 samples were collected from 19, 3, 5, 4, and 1 natural populations in Guizhou, Hubei, Hunan, Sichuan, and Yunnan, respectively.

**Figure 6:**
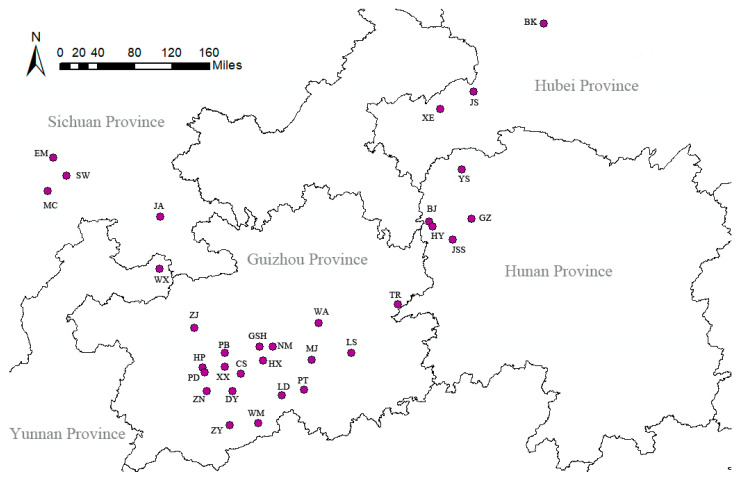
Sampling locations of natural populations of *C. bodinieri*.

Samples were collected by random sampling, and 200–300 g of leaf tissue was collected from individual plants. The tissue samples were then mixed, weighed evenly, and stored in an airtight container and used to record and collect geographic location information (Table 3, Figure 6). All collected plant materials were identified by Dr. Xie Yifei of Gannan Normal University and stored in the Nanling Herbarium.

### 4.2. Experimental Methods

#### 4.2.1. Distillation of Essential Oil

The essential oils were extracted and collected from each of the samples by reflux hydrodistillation. The leaf samples (200–300 g) were placed in the device (Reflux steam distillation apparatus) and purified water (sample weight/water weight = 1:10) was added. After 2 h of steam distillation, the essential oils were obtained by the liquid separator on the device. Water in the essential oils was removed with anhydrous sodium sulphate, and the weights of the essential oils were measured using an electronic balance. The extracted essential oils were dissolved in anhydrous ethanol (essential oil/ethanol = 30:970) and analysed using GC-MS and GC-FID.

#### 4.2.2. Essential Oil Chemical Composition Analysis

QP2020 GC-MS (Shimadzu, Kyoto, Japan) was used in this test. ASH-RXI-5SILMS column (30 m × 0.25 mm × 0.25 μm) was used as carrier gas with ultra-pure helium gas. Flow rate 1.0 mL/min, injection port temperature 200 °C, injection volume 1.0 μL, dplit ratio 20:1, EI ion source temperature 280 °C, scanning mass range 50–650 *m/z*. The initial temperature of GC was 60 °C for 2 min, and the GC was heated up at 5 °C/min to 220 °C and maintained for 20 min. The GC-MS data processing system was used to search the database (NIST 8.0), and a series of n-alkanes (C8-32, Sigma-Aldrich, St. Louis, MO, USA) retention indices under the same conditions were compared with the relevant literature to identify the compounds [39].

Quantitative analyses were performed using the GC-FID method on a GC-2010 Plus (Shimadzu, Kyoto, Japan) equipped with the same column as GC-MS. The conditions were the same as GC-MS except that N2 gas was used as carrier gas. GC data processing system was used to calculate the relative content of compounds. Relative content (%) = 100 × compound peak area/total peak area.

### 4.3. Data Analysis

The natural population distribution map of *C. bodinieri* was drawn using ArcGIS (Version 10.0; Esri, Redlands, CA, USA), the oil yield frequency was statistically analysed by Origin (Version 19.0; OriginLab, Northampton, MA, USA), and the chemical population distribution, principal component analysis (PCA), and chemical component correlation heat map were mapped using the ggplot and pca package analysis tools in R language. The correlation analysis of essential oil characteristics with environmental and climatic factors was performed using SPSS software (version 22.0; SPSS Inc, Chicago, IL, USA)

## 5. Conclusions

Sixteen chemotypes were observed in the essential oil from Chinese *C. bodinieri* leaves, among which the camphor-type, citral-type, and eucalyptol-type were dominant and found in the largest number of individuals. Certain chemotypes, such as the trans-nerolidol-type, borneol-type, cymol-type, elemol-type, methyl isoeugenol-type, and selina-6-en-4-ol-type, were first reported in *C. bodinieri*, and these results can provide a basis for studying the biosynthesis mechanism of *Cinnamomum* essential oil and breeding improved varieties. The average oil yield of *C. bodinieri* leaves was 0.75%, and the oil yield among individuals showed a difference of more than 400 times. The oil yields of the GZ-WA, GZ-DY, and GZ-LS communities were the highest, with an average yield of more than 1.5%. In addition, the yield and main component contents of *C. bodinieri* leaf essential oil were mainly affected by altitude, sunshine duration, and temperature. Low-altitude and warm climate areas are more suitable for the synthesis of essential oil in this species.

## Figures and Tables

**Table 1:** Basic information of oil yield of different chemical types of *C. bodinieri*.

NO	Chemotype		N	Ratio (%)	EO Mean Yield (%)	EO Min. Yield (%)	EO Max. Yield (%)	Principal Component Content (%)
1	camphor		333	37.63	1.18 ± 0.87 ^a^	0.02	4.28	30.42–98.52
2	citral		160	18.08	0.26 ± 0.22 ^e^	0.02	1.51	31.77–82.91
3	eucalyptol		101	11.41	0.73 ± 0.58 ^b^	0.02	2.86	41.20–67.09
4	linalool		82	9.27	1.06 ± 0.74 ^ad^	0.02	2.73	31.39–97.14
5	trans-nerolidol		65	7.34	0.31 ± 0.28 ^ce^	0.02	1.62	30.07–93.20
6	mix		73	8.25	0.29 ± 0.32 ^ce^	0.02	1.98	/
7	Other	α-cadinol	9	1.02	0.16 ± 0.07 ^ce^	0.07	0.33	30.01–62.79
8		α-phellandrene	1	0.11	0.77	/	/	33.32
9		borneol	2	0.23	0.27 ± 0.01 ^bcde^	0.26	0.28	32.91–35.86
10		cedrenol	4	0.45	0.27 ± 0.07 ^bce^	0.18	0.38	30.79–36.54
11		cymol	10	1.13	0.28 ± 0.26 ^ce^	0.09	1.04	31.68–67.71
12		elemol	23	2.60	0.30 ± 0.30 ^ce^	0.01	1.45	33.58–74.29
13		methyleugenol	8	0.90	0.64 ± 0.59 ^bcde^	0.10	2.09	61.12–94.86
14		methylisoeugenol	12	1.35	0.65 ± 0.53 ^bc^	0.14	2.30	49.45–88.81
15		safrole	1	0.11	0.08	/	/	77.09
16		selina-6-en-4-ol	1	0.11	0.95	/	/	67.15

Note: ^”a,b,c,d,e”^ means in the same column without a common superscript difference (*p* < 0.05).

**Table 2:** Correlation between the essential oil components of *C. bodinieri* leaves and environmental factors.

Compounds	Latitude	Longitude	Altitude/m	Annual Average Temperature/°C	Annual Rainfall/mm	Annual Average Sunshine Duration/h
Essential oil yield	−0.29	0.250	−1.090 **	0.103 **	0.91 *	0.045
Camphor	−0.045	0.086	−0.137 *	0.061	−0.079	0.134 *
Eucalyptol	0.177	−0.149	−0.206 *	0.177	0.120	−0.019
Linalool	−0.069	0.076	−0.229 *	0.052	0.166	−0.197
Elemol	0.240	−0.471 *	0.117	−0.036	−0.110	0.631 **
Citral	−0.002	0.033	−0.310 **	0.226 **	0.084	0.014
Trans-nerolidol	−0.101	0.055	0.393 **	0.044	−0.296 *	−0.007
Cymol	−0.375	0.131	−0.458	0.228	0.067	0.555 *

Note: ** The correlation was significant at the level of 0.01. * The correlation was significant at the level of 0.05.

**Table 3:** Natural population survey and sample collection site information of *Cinnamomum bodinieri*.

No.	Code	Sampling Location	Number of Samples	Geographical Coordinates		Altitude/m
				Latitude (N)	Longitude (E)	
1	GZ-CS	Changshun County, Guizhou Province	28	26.177239	106.3958	1229–1264
2	GZ-DY	Duyun County, Guizhou Province	33	25.925159	107.403076	756–831
3	GZ-GSH	Guanshanhu District, Guizhou Province	29	26.603312	106.688876	1098–1188
4	GZ-HX	Huaxi District, Guizhou Province	26	26.385045	106.74266	987–1084
5	GZ-LD	Luodian County, Guizhou Province	38	25.392029	106.672442	368–424
6	GZ-LS	Leishan County, Guizhou Province	27	26.504957	108.159398	765–872
7	GZ-MJ	Majiang County, Guizhou Province	32	26.402643	107.530591	846–886
8	GZ-NM	Nanming District, Guizhou Province	26	26.605626	106.901249	1108–1193
9	GZ-PB	Pingba County, Guizhou Province	26	26.509908	106.137244	1174–1242
10	GZ-PD	Puding County, Guizhou Province	31	26.271244	105.782499	1351–1384
11	GZ-PT	Pingtang County, Guizhou Province	28	25.835329	107.044541	660–1096
12	GZ-TR	Tongren City, Guizhou Province	10	27.287726	108.897560	812–870
13	GZ-WA	Wengan County, Guizhou Province	11	26.984027	107.635491	763–916
14	GZ-WM	Wangmo County, Guizhou Province	12	25.354239	106.215592	1060–1270
15	GZ-XX	Xixiu District, Guizhou Province	30	26.283751	106.133002	1230–1282
16	GZ-HP	Huangping County, Guizhou Province	29	26.193944	105.816504	1324–1409
17	GZ-ZJ	Zhijin County, Guizhou Province	25	26.911583	105.650365	1289–1394
18	GZ-ZN	Zhenning County, Guizhou Province	29	25.901472	105.851612	934–1093
19	GZ-ZY	ZiYun County, Guizhou Province	41	25.901472	106.256148	873–1236
20	HB-BK	Baokang County, Hubei Province	21	31.768400	111.234308	561–714
21	HB-JS	Jianshi County, Hubei Province	24	30.401166	109.582340	560–873
22	HB-XE	Xuanen County, Hubei Province	17	30.684367	110.112064	681–844
23	HN-BJ	Baojing County, Hunan Province	28	28.611383	109.39728	308–863
24	HN-GZ	Guzhang County, Hunan Province	41	28.650150	110.077720	412–767
25	HN-HY	HuaYuan County, Hunan Province	34	28.532613	109.454847	432–508
26	HN-JSS	Jishou City, Hunan Province	25	28.317948	109.783346	246–286
27	HN-YS	Yongshun County, Hunan Province	30	29.436850	109.922745	478–519
28	SC-JA	Jiangan County, Sichuan Province	27	28.679040	105.100912	315–402
29	SC-EM	Emeishan City, Sichuan Province	30	29.58042	103.44652	421–464
30	SC-SW	Shawan District, Sichuan Province	33	29.33298	103.61326	386–342
31	SC-MC	Muchuan County, Sichuan Province	30	29.15152	103.39304	378–482
32	YN-WX	Weixing County, Yunnan Province	34	27.842589	105.166708	1120–1148
Total			885			

## Data Availability

All the data are shown in the main manuscript.

## Sample Availability

Not available.
